# Waveguiding and SERS Simplified Raman Spectroscopy on Biological Samples

**DOI:** 10.3390/bios9010037

**Published:** 2019-03-03

**Authors:** Immanuel Valpapuram, Patrizio Candeloro, Maria Laura Coluccio, Elvira Immacolata Parrotta, Andrea Giugni, Gobind Das, Gianni Cuda, Enzo Di Fabrizio, Gerardo Perozziello

**Affiliations:** 1 Department of Experimental and Clinical Medicine, University “Magna Graecia” of Catanzaro, 88100 Catanzaro, Italy; immanuelloyola@gmail.com (I.V.); patrizio.candeloro@unicz.it (P.C.); coluccio@unicz.it (M.L.C.); parrotta@unicz.it (E.I.P.); cuda@unicz.it (G.C.); 2 Structural Molecular Imaging Light Enhanced Spectroscopies Laboratory, Physical Science and Engineering Division, King Abdullah University of Science and Technology, Thuwal 23955-6900, Saudi Arabia; andrea.giugni@kaust.edu.sa (A.G.); gobind.das@kaust.edu.sa (G.D.); enzo.difabrizio@kaust.edu.sa (E.D.F.)

**Keywords:** prism coupling, optical waveguide, optical biosensors, Raman micro-spectroscopy

## Abstract

Biomarkers detection at an ultra-low concentration in biofluids (blood, serum, saliva, etc.) is a key point for the early diagnosis success and the development of personalized therapies. However, it remains a challenge due to limiting factors like (*i*) the complexity of analyzed media, and (*ii*) the aspecificity detection and the poor sensitivity of the conventional methods. In addition, several applications require the integration of the primary sensors with other devices (microfluidic devices, capillaries, flasks, vials, etc.) where transducing the signal might be difficult, reducing performances and applicability. In the present work, we demonstrate a new class of optical biosensor we have developed integrating an optical waveguide (OWG) with specific plasmonic surfaces. Exploiting the plasmonic resonance, the devices give consistent results in surface enhanced Raman spectroscopy (SERS) for continuous and label-free detection of biological compounds. The OWG allows driving optical signals in the proximity of SERS surfaces (detection area) overcoming spatial constraints, in order to reach places previously optically inaccessible. A rutile prism couples the remote laser source to the OWG, while a Raman spectrometer collects the SERS far field scattering. The present biosensors were implemented by a simple fabrication process, which includes photolithography and nanofabrication. By using such devices, it was possible to detect cell metabolites like Phenylalanine (Phe), Adenosine 5-triphosphate sodium hydrate (ATP), Sodium Lactate, Human Interleukin 6 (IL6), and relate them to possible metabolic pathway variation.

## 1. Introduction

Many alterations, though minimal, in biological structures or cell physiology of body’s organs, leave traces in the chemical composition of the blood or other body fluids, like serum and saliva. By analyzing differences between physiological and altered states, serious illnesses can be diagnosed and administered [1,2,3]. A specific molecular signature can be traced by investigating the fluid, in particular biomarkers that, representing a gauge of normal or aberrant biological response (pathogenic or pharmacologic), allow the early stage mutations identification, revealing the status of the entire body. Hence, biomarkers play a vital role in the early diagnosis in, for instance, cancer or any other disease, in pathology progression, in targeted drug delivery and in the response to therapies [4,5,6,7]. Considering the primary need for ultrasensitive, ease of use and fast detection methods, new classes of biosensors are gaining increasing attention [8,9]. 

Most often, well-established detection approaches require huge and complex setups to reach adequate sensitivity and analytical resolution to reveal small traces of biomarkers [10,11,12]. In addition, conventional analyses usually require complex samples pretreatment aiming at sorting the components of a biological specimen. In order to overcome the above limitations, sensitive, fast, easy and a low cost assaying system are continuously investigated in order to improve the current technologies with their transduction and detection mechanisms [13,14,15]. 

In this contest, Raman spectroscopy represents a viable alternative methodology when integrated, with an advanced device that offers spatial and compositional selectivity and sensitivity. The technique itself relies on the analysis of the light spontaneously scattered from the specimen allowing for chemical compound analysis. From the scattered spectrum, it is possible to extract chemical information associated with the vibrational modes of the molecules. Indeed, each molecule shows up a unique Raman spectrum so that a specific fingerprint can be associated with each biological substance, a fact that is of fundamental importance to establish the specificity of the analysis method [16,17]. Differently from many other analytical techniques, Raman spectroscopy allows label-free analysis of samples and it is compatible with measurements in aqueous solutions, indispensable conditions for reducing the biological sample pretreatment [18,19,20,21,22,23]. 

However, if the sample is complex, in terms of molecular species and their concentration variability, Raman spectroscopy faces major limits in resolving each component at the same time due to vibrational bands overlaps when the mixture contains several kinds of molecules. In addition, a known disadvantage of the spontaneous Raman effect is about the signal intensity due to a low Raman scattering cross section ~10^−24^–10^–28^ cm^2^. Another possible limitation is the minimum accessible volume, determined by the optical diffraction limit, of about 300 nm. 

To overcome these limitations, it is possible to employ Raman spectroscopy technique in combination with specific devices, like a SERS surface or a proper designed nanoplasmonic device [24,25]. In such devices, realized by noble metals, an exciting laser source couples with specific plasmons of the structures (localized or propagating) that in turn produce a quite strong local enhancement of the incident field and the scattering from nearby emitters. In the SERS technique, an extremely diluted sample is deposited on the roughened metal surface so that only a few molecules of the analyte will be in the enhanced near field range produced by the plasmonic structure (few to tens on nm). The possibility offered by the last generation of nanofabrication equipment to fabricate and control metal nanostructures at the nanometric level makes possible the realization of complex plasmonic nano devices able to solve highly diluted complex mixtures and that in turn become ideal candidates for mass production of a new generation of biosensors [26,27,28].

The potentiality of plasmonic nanodevices is often hampered by other experimental requests, for example imposed by the optical set-ups or by the specific sample holder needed for Raman measurements. Limitations became crucial especially when living organisms need to be analyzed in specific environments (thermo-regulated chambers, channels, flasks, vials, microfluidic devices). Due to the specific bio-physics application or device configuration, it is common that the suited high numerical aperture objective cannot be used because, for example, the substrates are thicker than the objective working distance or because the required materials absorb and scatter excessively the optical excitation. Therefore, for those and similar cases, there is a strong demand for new sensors, designs and/or new experimental layouts to pursue the task of simplified biological analysis. Several scientific groups have used different methods to optimize optical configurations to perform enhanced Raman spectroscopy. Mayer et al. [29] developed an optical platform to combine SPR (surface plasmon resonance) and SERS microscopy in the Kretschmann configuration. Recently Petrin has developed analytical calculations to elucidate and optimize such well-known configuration [30,31]. Huang et al. [32] used plasmonic waveguides to excite SERS. However, driving optical signals in locations, difficult to be accessed by a conventional optical microscope, remain an issue. In this sense, using optical waveguides in combination with a prism coupler [33] and nanoplasmonic structures give more flexibility to the standard optical configurations.

In this work, we propose a sensor for non-confocal excitation of the Raman scattering measurement in which the SERS surface is excited by the evanescent field of a laser propagating along a specific OWG located just below the plasmonic structures (the sensing surface). An optical prism couples the laser source to the OWG [34,35]. We fabricate the OWGs and the SERS surfaces on the same flat glass slide by using a simple fabrication process based on optical lithography and etching processes. The proposed configuration has the advantage of a mechanically stable structure that, driving the light in a specific point of the substrate by means of an optical waveguide allows the decoupling of the near field excitation from the far field collection and avoiding possible geometric or space limitations related to the external optics. We remark that an equivalent optical configuration also works to collect the signal from the SERS surface without the use of an optical microscope [36,37]. In addition, the use of OWGs allow for designing sensors in which the excitation and emission follow different optical paths, allowing for reducing the background signal consistently and hence increase the signal-to-noise ratio. This will also reduce the use of optical filters along the collection optics. 

As a general comment, high-quality Raman measurements from thin/transparent samples require the use of specific substrate materials to reduce background signals. The most used are calcium fluoride and quartz, both characterized to have ideally negligible broadband signals, even considering the bulk materials. The use of OWG is in line with these requirements. In particular, the proposed OWG has a core of indium tin oxide, ITO, covered by the plasmonic structures in the sensing area, a cladding of “air” on the upper and lateral sides, and “glass” on the bottom side. An advantage of using an ITO OWG relies also on the fact that ITO is conductive and can be used as a substrate in electronic beam lithography to fabricate nano plasmonic structures used as a SERS surface.

The simplest SERS device can be obtained simply generating a nano-rough surface by a selective etching process followed by a plasmonic material coating whose thickness is in the range of 30–50 nm.

A further step in the functional complexity and in the analytical performances of the device can be obtained by fabricating on the sensing area with a spatial control of the nano plasmonic structures. Here, we propose an array of nano-dimers, spatially arranged in a square grid, with reciprocal distances of about one μm. With such device, it is possible to excite and spatially resolve the enhanced Raman signal by conventional optics, simply focusing the collection on each nanostructure (or acquiring the full sample images in hyperspectral Raman configuration, in this case, the wavelength is scanned step by step while images preserve the spatial information). The signal detected arises only from the molecules inside the hotspots generated in between the dimers. A typical size of the gap is in the 10 nm range. The scattering volume results in being naturally confined well below the diffraction limit, and, due to the evanescent nature of the plasmonic field, the main contribution to the signal arises from the molecules in the nanodimers gap. Due to localization of the enhancement in the gap, a hot spot, the probability to detect more than one or very few type of molecules from a complex mixture is very low, thus the nano-dimers device, reducing in this sense the complexity, acts as a sorter of the solution. We proposed such an array to perform biological analysis of complex mixtures without the need to pretreat the sample by separating the components. In addition, each nanostructure can be used as a pixel, where the total number of pixels can be put in correlation to the solution composition and to concentration of each species present in the solution [27].

In this work, we demonstrate an array of nanodimers, specifically used to quantify cell metabolites in a solution. In particular, Adenosine 5-triphosphate sodium hydrate (ATP), Sodium Lactate, and Human Interleukin 6 (IL6) were detected, whose concentration can be connected to cell metabolism disorders of cancer cells and can be used as biomarkers in cancer therapies [38,39].

## 2. Materials and Methods

### 2.1. Materials

The Glass substrates (2 × 3 cm) covered with indium tin oxide (ITO) were purchased from Crystan Ltd. (Poole, UK). The silicon wafers (Si, <100>, P-doped, Res 1–10 Ohm-cm) were purchased from Jocam SRL (Milano, Italy). The positive photoresist S1813 and the developer MF-322 were purchased from All Resist GmbH (Strausberg, Germany). Hydrocloridric acid (HCl) 37 wt. %, acetone 99 wt. %, Hydrofluoridric acid (HF) 48 wt. %, Iso Propyl Alcohol (IPA) 99 wt. %, anisole 99 wt. %, gold trichloride (AuCl_3_) were purchased from Sigma Aldrich (Merck KGaA, Darmstadt, Germany). Au for the sputtering systems was purchased from Alfaaesar (Thermo Fisher (Kandel) GmbH, Karlsruhe, Germany). PMMA A2 was purchased from MicroChem (Westborough, MA, USA). Phenylalanine (Phe) was purchased from Sigma Aldrich (Merck KGaA, Darmstadt, Germany, CAS Number 63-91-2) and used to characterize the SERS surface fabricated on the OWG. ATP (CAS number 34369-07-8), Sodium Lactate (CAS number 867-56-1)) were purchased from Sigma Aldrich (Merck KGaA, Darmstadt, Germany), IL6 was purchased from BD Bioscience (San Josè, CA, USA), Human IL6 standard, 558464, Cat 51-9003499) and used to characterize the nanoplasmonic devices.

### 2.2. Working Principle

**Figure 1:**
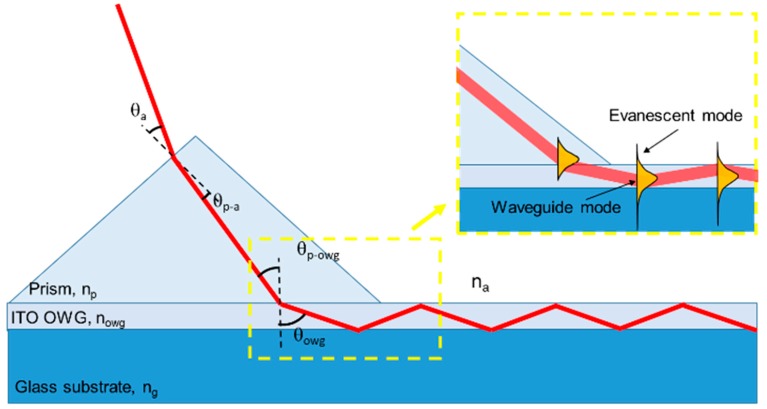
Illustration of propagation of laser through the prism and the OWG, and a schematic representation of the evanescent field propagation (*n_p_* > *n_owg_* > *n_g_* > *n_a_*).

The presented biosensor, schematically represented in Figure 1 (left), consists of a glass substrate an ITO OWG fabricated on the topside along the longitudinal direction that connects the two opposite edges. A SERS active surface is then realized on one of the OWG side, while a prism coupler is placed on top of the other. With such configuration, a laser source is directed toward the prism at a certain angle (Figure 1(right)) and coupled to the OWG thanks to the proper choice of the optical path and to the refractive indexes of the materials. Then, the light, travelling through the OWG, reaches the plasmonic surface originating the SERS phenomena. In order to characterize the SERS active area covered by biomolecules, we tested it with a microscope in a confocal configuration.

### 2.3. Prism Coupling

A *λ*/4 Rutile TiO_2_ prism (Thorlabs GmbH, Newton, MA, USA, 45-45-90, size 5 × 5 × 5 mm) having a refractive index (*n_p_* = 2.6), higher than the refractive index of the ITO (*n_owg_* = 1.82), was used to couple part of the power contained in the laser beam source to the thin ITO OWG (350 nm). Such a method does not need sub-micrometer alignment of the beam to the edge of the OWG, or the need for matching the numerical aperture of the illumination optics to the film. The laser beam can have a diameter tens of times that of the OWG when coupled by means of the prism, and still a consistent fraction of light power will enter the OWG.

In particular, for external incidence smaller than a particular angle, *θ*_max, the propagating beam at the prism interface tunnels into the ITO film that acts as an OWG over the glass substrate (*n_g_* = 1.42). When tunneling occurs, phase matching conditions are required between the propagation constant of the *m*th mode in the OWG (*β_mowg_*) and the incident light traveling in the prism (*β_mp_*) at an angle $\theta_{p-owg}$ normal to the OWG surface.

This means that:

(1) $$\beta_{mowg}=\beta_{mp}\to kn_{owg}\sin\theta_{owg}=kn_{p}\sin\theta_{p-owg},$$

where *k* = 2π/*λ*_0_ is the wave vector and $\theta_{owg}$ is the angle between the light propagating into the OWG and the normal to the OWG surface.

In addition, for a lossless propagation into the OWG, total internal reflection should be ensured between the OWG, the glass substrate and air. For the purpose, the tight constraint comes from the interface characterized by the smaller refractive index jump, the ITO OWG-Glass. In this case, the Snell’s law is expressed as:

(2) $$n_{owg}\sin\theta_{owg}=n_{g}\sin\theta_{g},$$

where $\theta_{g}$ is the angle between the light refracted from the OWG into the substrate and the normal to the OWG surface. Total internal reflection is ensured if $\sin\theta_{g}=1$, which means that:

(3) $$\theta_{owg}=\sin^{-1}\left(\frac{n_{g}}{n_{owg}}\right)=51.26{}^{\circ}.$$

Using this value in Equation (1), we can calculate:

(4) $$\theta_{p-owg}=\sin^{-1}\left(\frac{n_{owg}\sin\theta_{owg}}{n_{p-owg}}\right)=33.12{}^{\circ}.$$

The light traveling in the prism creates an angle at the interface between air and prism normal to its surface (this interface is at an angle of 45° from the OWG surface):

(5) $$\theta_{p-a}=45{}^{\circ}-\theta_{p-owg}=11.88{}^{\circ}.$$

To ensure total internal reflection, the propagating angle should be less than 11.88° and can be put in relation with the angle $\theta_{a}$ of the incident light traveling from the laser beam with respect to the normal from the prism surface by using again the Snell’s law at the interface between air and prism:

(6) $$n_{p}\sin\theta_{p-a}=n_{a}\sin\theta_{a}\to \theta_{a}=\sin^{-1}\left(\frac{n_{p}\sin\theta_{p-a}}{n_{a}}\right)=32.36{}^{\circ}.$$

This value represents the maximum angle, *θ_a_*_max, between the incident laser beam and the normal to the prism surface to ensure total internal reflection into the OWG and so to ensure that the light is guided into the OWG.

It can be noticed that, without the prism, the light could not be guided directly into the OWG because applying the Snell’s law in this case

(7) $$n_{owg}\sin\theta_{owg}=n_{a}\sin\theta_{a}\to \sin\theta_{a}=\left(\frac{n_{owg}\sin\theta_{owg}}{n_{a}}\right).$$

Obviously,

(8) $$\sin\theta_{a}\leq 1.$$

Therefore,

(9) $$\left(\frac{n_{owg}\sin\theta_{owg}}{n_{a}}\right)\leq 1\to \theta_{owg}\leq \sin^{-1}\left(\frac{n_{a}}{n_{owg}}\right)\leq 33{}^{\circ},$$

which does not satisfy the condition that this angle should ensure total internal reflection as calculated in Equation (3).

Moreover, there is a minimum value of $\theta_{a}$ which could ensure that the laser beam is coupled in the OWG, which corresponds to an angle $\theta_{owg}=90{}^{\circ}$.

For $\theta_{owg}=90{}^{\circ}$, using Equation (4), it can be found that $\theta_{p-owg}=44.45{}^{\circ}$, and, applying Equation (5), it is $\theta_{p-a}=0.55{}^{\circ}$, that, put in Equation (6), corresponds to $\theta_{a}=1.43{}^{\circ}$.

In conclusion, $1.43{}^{\circ}\leq \theta_{a}\leq 32.36{}^{\circ}$ represents the range for ensuring prism coupling.

### 2.4. Optical Waveguides

**Figure 2:**
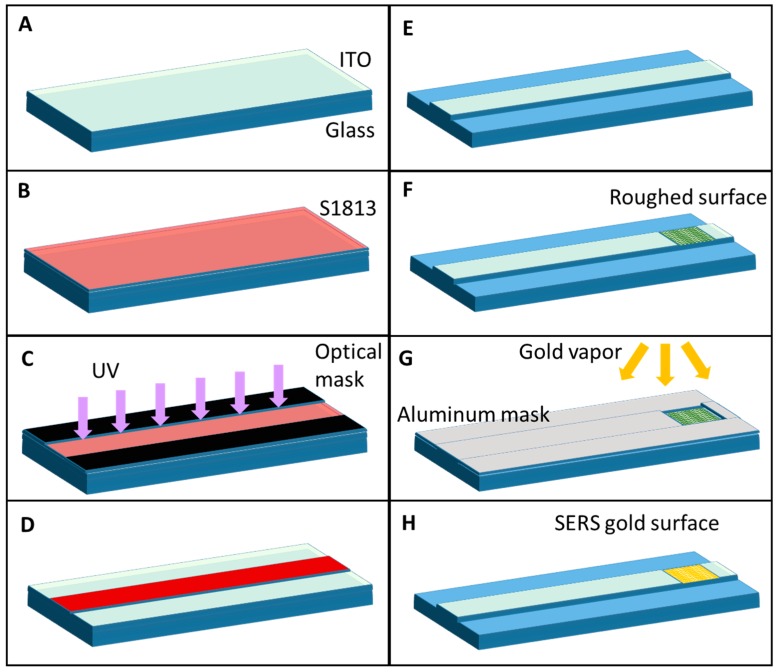
Schematic representation of the fabrication process: (**A**) glass + ITO substrates are clean; (**B**) the positive photoresist S1813 is spun on the substrate; (**C**) the sample is exposed by UV light under an optical mask; (**D**) the exposed resist is developed; (**E**) the ITO is wet-etched to create the OWG; (**F**) the ITO is roughened on one side of the OWG; (**G**) the sample is exposed to gold physical vapor deposition under an aluminum mask to create the SERS surface on top of the ITO roughened surface; (**H**) final device.

The device was fabricated by the photolithographic process (Karl Suss Mask Aligner MA 45, SussMicroTec GA, Garching, Germany) illustrated in Figure 2 following specific steps. First, a positive photoresist S1813 was spun on a glass substrate (20 mm × 30 mm × 0.17 mm) coated with 350 nm thickness of ITO at spin velocity of 4000 rpm for 60 s (Figure 2B) followed by baking at 90 °C for curing. Subsequently, it was subjected to UV light exposure for 26 s, protected by an optical mask reproducing the layout of the OWG (Figure 2C). Post exposure, the S1813 was developed using a developer (MF-322) for 40 s and rinsed with deionized water (DI) to remove the unexposed part. The ITO OWG is then etched for 60 s using HCl, and acetone is used to remove the residual S1813 to reproduce the geometrical pattern of OWG (Figure 2D,E). In the end, the OWG has a rectangular cross-section 5 mm wide and 350 nm high and it is 3 cm long. On one side of ITO OWG, the surface is roughed (Ra = 150 nm) manually by using an abrasive paste (Figure 2F). Finally, gold (Au) was sputtered on the ITO for 30 s after having protected it with an aluminum foil used as a mask to reproduce the SERS surface at the end of the OWG in correspondence of the roughed surface (Figure 2G,H).

### 2.5. Plasmonic Nanodimers

A periodic pattern of nanodimers could be fabricated on top of the OWG. In this case, we realized a plasmonic resonator by gold dimers constituted by two circles of about 100 nm in diameter with a nominal gap of 10 nm. These were fabricated by electron beam lithography and electroless deposition [40] employing patterns of matrices (4 × 15) composed of pairs of circles. 

**Figure 3:**
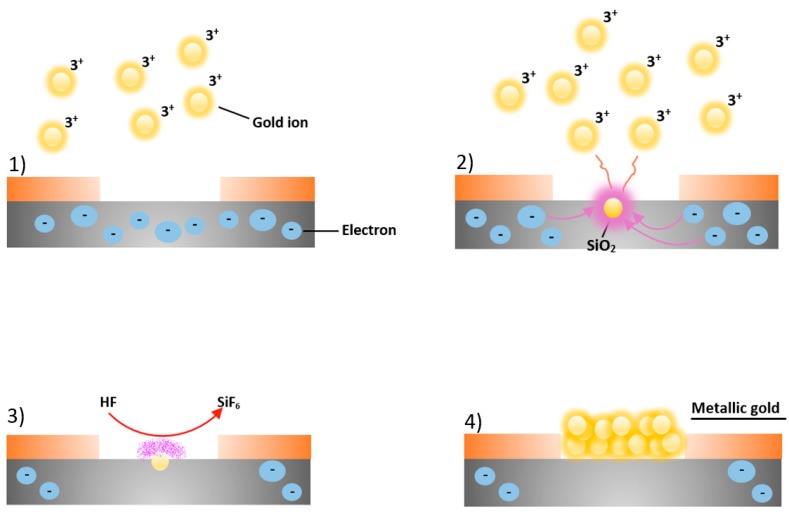
Schematic representation of electroless deposition of gold nanoparticles. (**1**) Au^3+^ ions in the solution near the silicon (Si) surface (in grey), in the proximity of the nanoholes patterned in the resist (in orange), capture electrons from the Si valence band; (**2**) a redox reaction starts between gold ions and Si, evolving in the Au^3+^ reduction into metallic gold nanoparticles and the oxidation of silicon into SiO_2_; (**3**) the hydrofluoric acid in solution reacts with the silicon oxide inducing the etching of SiO_2_ and the dissolution of it as SiF_6_^2-^ (violet cloud); (**4**) the Au nuclei deposited continue to attract electrons from bulk silicon, working as a catalytic surface for the reduction of new Au^3+^ ions until the desired thickness is reached.

The electron beam lithographic processes were executed in the clean room at the Bionem laboratory (University Magna Graecia of Catanzaro) by using the high-resolution Electron beam lithography system CABLE 9000C. For characterization purposes, the electron lithography was performed on Silicon wafers cut 2 cm × 4 cm, using a diamond tip. The resultant pieces were immersed in boiling acetone, rinsed in hot IPA to favorite a better adhesion of the resist and then dried with N_2_. The substrates were coated with PMMA A2 in anisole, which is a high resolution positive, resist. The coating process was realized by spinning the samples at 0.4 bar for 60 s at 5000 rpm and baking them on a hot plate for 2 min at 170 °C. The thickness achieved for the layer of resist was 50 nm. The samples were then patterned by electron beam lithography at 30 KeV with a current of 100 pA and an exposure dose of 2500 µC/cm^2^. Finally, the pattern was developed in IPA at 4 °C for 60 s in order to have nanoholes, reproducing the negative of the nanodimers. The silicon wafers were then immersed in HF with the purpose of removing the layer of silicon oxide. The result is a hydrogenated silicon surface inert to reactions with compounds such as O_2_, CO_2_, CO, etc., and thus available to subsequent stages of deposition. Electroless deposition of the gold in the nanoholes was performed by immersing the substrates first in a solution 0.10 mM of AuCl_3_ and 0.15 M of HF for 20 s and then in ionized water to halt the reactions. The electroless deposition process is shown in Figure 3.

The chemical process consists of the following two half reactions:
$\mathrm{Si}+2H_{2}O\to {\mathrm{SiO}}_{2}+4H^{+}+4e^{-}$AnodeSilicon oxidation,${\mathrm{Au}}^{3+}+3e^{-}\to {\mathrm{Au}}^{0}$CathodeGold reduction.

The Standard redox potentials of reactions are −0.9 V and 0.8 V.

### 2.6. Morphology Characterization

SEM images of the nanodevices were captured using a Dual Beam (SEM-FIB)-FEI Nova 600 Nano Lab system (Thermo Fisher Scientific, Hillsboro, OR, USA). During the acquisitions, beam energies of 5 and 15 keV, and corresponding electron currents of 0.98 pA and 0.14 nA were used. In some cases, the mode 2 configuration was used, whereby images can be magnified over 2500k× and ultrahigh resolution may be achieved. In this modality, the immersion lens was switched on.

### 2.7. Raman Equipment Set-Up and Measurements

The biological samples at a concentration of 10 μM in DI water were deposited on the SERS surface and on the plasmonic dimers device and there evaporated. In particular, Phenylalanine was deposited on the SERS surface, while a mixture of Adenosine 5-triphosphate sodium hydrate (ATP), Sodium Lactate and Human Interleukin 6 (IL6) was deposited on the nanodimers.

A Raman micro-spectroscopy equipment “Alpha 300RA” from WITec GmbH was used to investigate the device performances.

For the characterization of the SERS surface excited through the OWG, an HeNe laser source (633 nm) coupled through a monomodal optical fiber was collimated by an optical fiber coupler (Achromatic FiberPort, FC/APC, f = 4 mm, 400–700 nm, Ø0.65 mm Waist, PAFA-X-4-A from Thorlabs GmbH) to the prism coupler (*λ*/4 Rutile TiO_2_ prism). The incident angle of the laser was set at the proper angle using a Compact 5-Axis Goniometer (PY005 from Thorlabs GmbH) having the attention to minimize reflection losses to guide into the ITO OWG the largest possible excitation for the SERS surface [15]. For the characterization of the nanodimers deposited on a microscale grid, a confocal excitation configuration was used. The HeNe laser was focused by a 100×/0.95 NA objective on different points to collect the spectra of Adenosine, ATP and IL6. The laser power at the sample was kept in the range of 0.1–0.5 mW. Indeed, due to SERS effect, larger laser power could result in a photo damaging of the probed sample. The backscattered light was collected through the same objective, filtered out of the Rayleigh peak by a notch filter, and sent to an 1800 lines per mm grating for spectral analysis. The probed spectral interval was from 1200 cm^−1^ up to 1800 cm^−1^, while the integration time range was between 0.5 and 2.0 s. The experiments were repeated three times and the spectra were then averaged. Raman spectra were recorded for the biological samples and compared with the standard spectra as can be obtained by conventional Raman spectroscopy of the sample deposited over silicon substrate surface, using equivalent concentrations. 

## 3. Results and Discussion

### 3.1. Device Morphology

**Figure 4:**
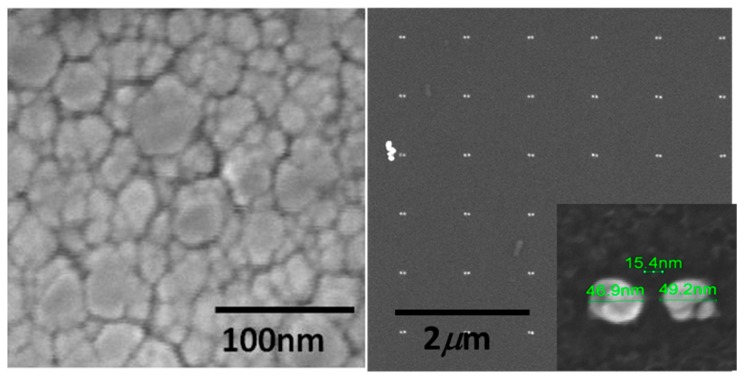
Left: SEM picture of the gold sputtered SERS surface; Right: SEM image of an array of dimers, and magnified picture of the nanodimer.

In Figure 4 (left), it can be observed the structures of the roughed SERS surface sputtered with gold. The characteristic granular structures are responsible for the optical enhancement. However, such structures are irregular and different in shape and dimensions implying an inherent difficulty to obtain spatially homogeneous scattered intensities and repeatable measurements because the SERS effect depends directly by the local structural conformation. In addition, with such substrate, there could be possible limitations to resolve complex mixtures due to the spatial resolution/localization obtainable; however, they can be used efficiently for qualitative measurements. 

In Figure 4 (right), the nanodimers matrix is shown and, in the inset, a representative dimer, as fabricated by means of the electroless process. The grown Au layer is roughly 50 nm thick with a standard deviation of 10 nm. The nano-dimers ensure giant SERS enhancement during Raman spectroscopy analysis. In this case, the periodic arrangement ensures reliable measurements and the high spatial localization (in the gap) allows for measuring complex mixtures without pre-treatment, as explained in the previous sections. Finally, a similar configuration allowed quantitative measurements [27].

### 3.2. Measurements through the OWG 

**Figure 5:**
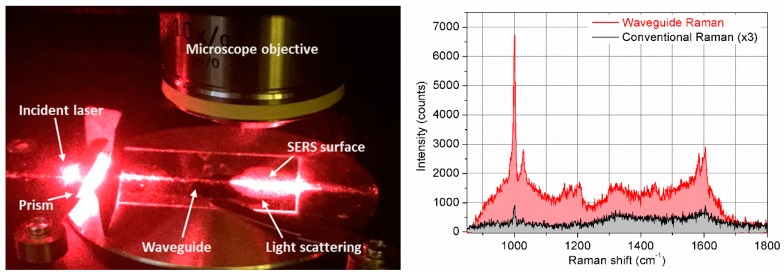
Left: Schematic representation of device layout: Optical set-up displaying the incident laser beam, the prism, the OWG, and the light scattering from the SERS surface (rough estimation of losses showed the value of optical losses >95%, which is in agreement with that published in literature [36], see Supplementary Materials); Right: Raman spectra of phenylalanine taken by conventional Raman spectroscopy (in black) and by using the developed device (in red).

Figure 5 (left) shows the experimental setup used in this experiment, where the light from an optical fiber is incident to the prism and, coupled through the OWGs, is scattered on the SERS surface. 

Our results (Figure 5, right) demonstrate that conventional Raman detects the main vibrations of phenylalanine (Phe), such as the peak at 1003 cm^−1^ from the ring breathing vibration, and the peak at 1032 cm^−1^ from an in-phase motion of C atoms [41]. A peak at 1207 cm^−1^ is assigned to side chain vibrations, whereas, at 1586 cm^−1^, it is basically assigned to the out-phase motion of N atoms, and the peak at 1606 cm^−1^ originates from the in-phase motion of C atoms of the phenyl ring. The combined OWG and SERS Raman is able to detect the main Phe peak at 1003 cm^−1^, the Raman bands around 1207 cm^−1^ and the peak at 1586 cm^−1^. Other intense peaks are observed at 1369 cm^−1^ and 1648 cm^−1^ as well due to SERS effect. It is well known in SERS experiments that the binding of biomolecules with Au nanoparticles could enhance the Raman signal coming from vibrations non-detectable with conventional Raman [42,43].

### 3.3. Measurements Collected from Nanodimers

**Figure 6:**
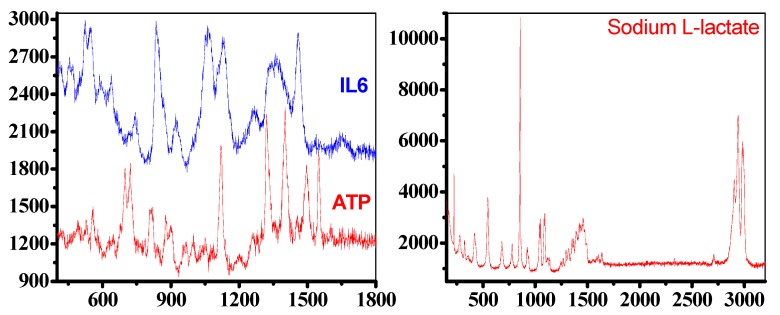
Left: Raman spectra for ATP (red) and IL6 (blue), measured between 500 and 1800 cm^−1^, Right: Raman spectra for Sodium L-Lactate, measure between 100 and 3300 cm^−1^, (information on the enhancement factor can be found in the Supplementary Materials).

The experimental results demonstrate that the nanodimers substrate (Figure 6) was able to detect the main Raman vibrations of the ATP. The peaks defining the molecular signature are at 1401 cm^−1^ from ring stretching vibration, 1318 cm^−1^ from stretching of three P=O bonds, near 1230 cm^−1^ from OH stretching vibration interacting with the overtone of in-plane POH bending vibration, 876 cm^−1^ and 719 cm^−1^ due to in-phase out-of-plane aromatic CH wagging vibrations of two and four adjacent hydrogens, respectively. It was also possible to detect the main vibrations of IL6, a strong peak at 524 cm^−1^ due to C double bonding with O rocking, 1060 cm^−1^ from aromatic amino acid residues, 1457 cm^−1^ from CH_2_ bending, 456 cm^−1^ due to C-N-C bend in amino acids and 1644 cm^−1^ due to NH_3_ deformation. To note that the Raman spectra for Sodium L-lactate shows an unusual profile constituted by the triplet at 2937 cm^−1^, 2982 cm^−1^ and 2905 cm^−1^ due to C-H vibrations, the peak at 853 cm^−1^ from single C-C single bond vibrations, peaks corresponding to the COO- functional group, at 221 cm^−1^ from COH deformation mode, at 543 cm^−1^ from CO_2_ wagging, at 1092 cm^−1^ due to asymmetric motion of branched carbon (C-C-C) and at 1053 cm^−1^ arising from C-CH_3_ stretching vibrations. In addition, a peak at 1459 cm^−1^ from asymmetric CH_3_ deformation modes is observed. 

## 4. Conclusions

We have successfully integrated and fabricated optical biosensors based on Raman Spectroscopy. Both systems proposed are compatible with Raman micro spectroscopy for label-free, high sensitive, rapid and continuous analysis of biological samples and cell biology studies.

In particular, the proposed biosensors offer new way to conduct Raman measurement introducing more flexibility in the excitation/collection scheme. A coupled SERS surface to the OWG benefits the deployment of evanescent wave excitation that is confined and limited to the sensing surface, thus reducing background signal, and attaining a high signal-to-noise ratio for the collected scattering. By using this device, in combination with a rough SERS surface, it was demonstrated that an enhanced Raman signal could be obtained on a phenylalanine sample. In addition, by using structured SERS surfaces with periodic patterns of plasmonic nanostructures, due to their high spatial localization of the hot spot, we measured individual spectral component from a complex samples without pretreatment. In this case, exploratory experiments were carried out on a sample of a mixture of ATP, Adenosine and IL6. The results suggest that it is possible to work with an integrated setup to obtain uniformity, reproducibility and consistency of biological measurements. This work supports the potentiality of Raman spectroscopy to detect metabolic fingerprinting in complex biosamples where the progression of a disease can be followed by the variation in biomolecular pathways through the different Raman pattern signals.

## Supplementary Materials

The following are available online at https://www.mdpi.com/2079-6374/9/1/37/s1. A rough estimation of optical losses can be estimated about 95%, due to coupling and propagation loss, which is in agreement with other published literature data [36]. The power loss in the waveguide was calculated by comparing peak intensities of phenylalanine measured in the device prism + OWG + SERS surface with the ones obtained on the SERS surface coupled directly by a microscope objective. In particular, the peak used for the comparison was that at 1606 cm^−1^ (in-phase motion of C atoms of the phenyl ring), which together to the peak at 1003 cm^−1^ (ring breathing vibration), is the most representative for that molecule. The enhancement factor (f) (or better, the Raman signal enhancement) compared to spntaneous Raman spectroscopy is 40, calculated as the intensity ratio between the ATP molecule peaks at 1401 cm^−1^ (ring stretching vibration) of SERS device (ISERS) and spontaneous (IRaman): f = ISERS/IRaman. The Electric field amplification is f^1/4^, as known from literature. For clarity’s sake, the spontaneous Raman spectrum is not reported preferring to evidence the SERS spectra of the three probe molecules.
