# Supplementary material for: Waveguiding and SERS Simplified Raman Spectroscopy on Biological Samples
Source: Biosensors (Basel). 2019 Mar 3;9(1):37. doi: 10.3390/bios9010037 (PMC6468818; doi:10.3390/bios9010037)
Supplement: Supplementary file 1 [file biosensors-09-00037-s001.pdf]

biosensors

MDPI

Supplementary Material

Waveguiding and SERS Simplified Raman Spectroscopy on Biological Samples

Immanuel Valpapuram [1], Patrizio Candeloro [1], Maria Laura Coluccio [1], Elvira Immacolata Parrotta [1], Andrea Giugni [2], Gobind Das [2], Gianni Cuda [1], Enzo Di Fabrizio [2], Gerardo Perozziello [1,*]

[1] Department of Experimental and Clinical Medicine, University "Magna Graecia" of Catanzaro, Catanzaro 88100, Italy; immanuelloyola@gmail.com (I.V.); patrizio.candeloro@unicz.it (P.C.); coluccio@unicz.it (M.L.C.); parrotta@unicz.it (E.I.P.); cuda@unicz.it (G.C.);

[2] Structural Molecular Imaging Light Enhanced Spectroscopies Laboratory, Physical Science and Engineering Division, King Abdullah University of Science and Technology, Thuwal 23955-6900, Saudi Arabia; andrea.giugni@kaust.edu.sa (A.G.); gobind.das@kaust.edu.sa (G.D.); enzo.difabrizio@kaust.edu.sa (E.D.F.)

* Correspondence: gerardo.perozziello@unicz.it

Raman Enhancement Factor

The enhancement factor (f) (or better, the Raman signal enhancement) compared to spntaneous Raman spectroscopy is 40, calculated as the intensity ratio between the ATP molecule peaks at 1401cm^{-1} (ring stretching vibration) of SERS device (I_{SERS}) and spontaneous (I_{Raman}): $f = I_{SERS}/I_{Raman}$. The Electric field amplification is $f^{1/4}$, as known from literature. For clarity's sake, the spontaneous Raman spectrum is not reported preferring to evidence the SERS spectra of the three probe molecules.

Power Loss Estimation

A rough estimation of optical losses can be estimated about 95%, due to coupling and propagation loss, which is in agreement with other published literature data [36].

The power loss in the waveguide was calculated by comparing peak intensities of phenylalanine measured in the device prism + OWG + SERS surface with the ones obtained on the SERS surface coupled directly by a microscope objective. In particular, the peak used for the comparison was that at 1606 cm^{-1} (in-phase motion of C atoms of the phenyl ring), which together to the peak at 1003 cm^{-1} (ring breathing vibration), is the most representative for that molecule.
